# HLA Association among Thai Patients with Diffuse and Limited Cutaneous Systemic Sclerosis

**DOI:** 10.3390/biomedicines12061347

**Published:** 2024-06-18

**Authors:** Worawit Louthrenoo, Nuntana Kasitanon, Antika Wongthanee, Yuko Okudaira, Asuka Takeuchi, Hiroshi Noguchi, Hidetoshi Inoko, Fujio Takeuchi

**Affiliations:** 1 Division of Rheumatology, Department of Internal Medicine, Faculty of Medicine, Chiang Mai University, Chiang Mai 50200, Thailand; nuntana.k@cmu.ac.th; 2 Department of Internal Medicine, Faculty of Medicine, Chiang Mai University, Chiang Mai 50200, Thailand; antika.wong@cmu.ac.th; 3 GenoDive Pharma Inc., Naka-cho Honatugi, Atsugi 243-0003, Japan; y-okuda@genodive.co.jp (Y.O.); hinoko@genodive.co.jp (H.I.); 4 Faculty of Economics, Sophia University, Tokyo 102-8554, Japan; asuka.takeuchi@sophia.ac.jp; 5 School of Pharmaceutical Sciences, University of Shizuoka, Shizuoka 422-8002, Japan; noguchi@u-shizuoka-ken.ac.jp (H.N.); fujio.tky@gmail.com (F.T.)

**Keywords:** systemic sclerosis, scleroderma, HLA, genetic association, haplotype

## Abstract

This study aimed to clarify the association of HLA Class I and II with dcSSc and lcSSc in Thais. HLA typing for 11 gene loci (Class I: HLA-A, B and C, and Class II [HLA-DR, DP and DQ]) was carried out using the Next Generation DNA Sequencing method (three fields) in 92 Thai patients with systemic sclerosis (55 dcSSc, 37 lcSSc) and 135 healthy controls (HCs). The distribution of HLA alleles in patients with dcSSc and lcSSc was compared. When compared with HCs, the AF of A*24:02:01, A*24:07:01, B*27:04:01 and B*27:06 showed an increasing trend in lcSSc patients without statistical significance. DRB1*15:02:01, DRB5*01:02:01, DQA1*01:01:01, DQB1*05:01:24, DPA1*02:01:01 and DPB1*13:01:01 increased significantly in dcSSc patients. DQB1*05:01:24 and DPB1*13:01:01 also increased significantly in lcSSc patients, but less significantly than in dcSSc patients. The association of DPB1*05:01:01 with lcSSc was significantly protective. HLA-A*24:02:01, B*27:06 and C*03:04:01 formed a three-locus haplotype that also constituted an eight-locus haplotype with DRB1*15:02:01, DQA1*01:01:01, DQB1*05:01:24, DPA1*02:01:01 and DPB1*13:01:01. There was a possibility that HLA Class I would play a role in the pathogenesis of lcSSc, while Class II played more of a role in the dcSSc in Thai patients.

## 1. Introduction

Systemic sclerosis is a systemic autoimmune disease characterized by the dysregulation of innate and adaptive immunity, leading to the production of autoantibodies, microvascular damage and fibrosis of the skin and visceral organs. The exact cause of systemic sclerosis remains unclear, but genetic and environmental factors have been implicated [1]. Although many genetic susceptibility genes have been identified in the pathogenesis of SSc, the human leukocyte antigen (HLA) gene has been studied most widely, and most studies have been related to HLA Class II molecules [2]. The contribution of HLA to SSc, particularly the HLA Class II molecules, has been determined in several ethnic groups, but the results are still controversial. For example, DRB1*11:01 was associated with American Caucasians [3], DRB1*11:01 and *11:04 with UK Caucasians [4,5], DRB1*11:04 with Europeans [6], DRB1*11:04 and DQA1*05:01 with Italians and DRB1*11:04 with Spaniards [7]; whereas DRB1*16:02 was associated with the Chinese [8], DRB1*15:02 and DRB5*01:02 with Koreans and the Japanese [9,10,11], and DRB1*15:02:01 and DRB5*01:02:01 with Thais [12].

Regarding HLA-DQ and DP, DQB1*03:01 was associated with Caucasian Americans, African-Americans and Hispanics [13], DQB1*03 with French Caucasians [14], DQB1*05:01 with the Chinese [15], DQB1*05:01 and DQB1*06:01 with the Japanese [10,16], DQB1*05:01:24 and DQA1*01:01:01 with Thais [12] and DQA1*05:01 with Italians [7]. DPB1*13:01 was associated with UK Caucasians’ SSc and Europeans [4,6] and European-Americans [5], DPB1*09:01 with the Japanese [10], and DPB1*13:01:01 with Thais [12].

Several studies have determined the contribution of HLA Class II molecules to the development of SSc. The charge of amino acid motifs at the 67–74 position on the third hypervariable region of HLA-DRB1 affected their association with SSc [17]. The uncharged polar residue tyrosine at 30, and amino acid sequence of ^71^TRAELDT^77^ on the first domain of the HLA-DQ β chain have been reported in SSc patients, being anti-topoisomerase antibody (ATA) positive [18]. Moreover, the amino acid motif ^67^FLEDR^71^ shared by HLA-DRB1 susceptible alleles was associated more highly with SSc patients who were ATA positive, when compared to the motif of ^71^TRAELDT^77^, shared by HLA-DQB1 susceptibility alleles [14]. In addition, the DQB1 alleles carrying polar glycine or tyrosine, but not leucine, at position 26 of the DQB1 first domain, also contributed to the pathogenesis of SSc [19]. 

In addition to a single HLA locus, which has been found to associate with SSc, several HLA loci form a haplotype that is associated with SSc. However, many of these studies have focused on the HLA Class II molecule. For example, DRB1*11:04, DQA1*05:01 and DQB1*03:01 constituted a haplotype among Caucasians and Hispanics [3], DRB1*08:04, DQA1*05:01 and DQB1*03:01 among African-Americans [3], DRB1*15:02 and DQB1*06:01 among the Japanese and Koreans [10,20], and DRB1*15:02:01, DQB1*05:01:24 and DPB1*13:01:01 among Thais [12]. 

Studies on the association of HLA Class I with SSc are limited and have shown conflicting results. HLA-B*08:01 is known to be a susceptible allele among Europeans, the Mexican Mestizo and Spanish SSc patients [6,21]. In contrast, B*44:03 is the protective allele among European Americans [5], C*16:01 among Australians and North Americans (United States) [22], and C*07:02 among the Mexican Mestizo [21]. In terms of haplotypes, C*07:01-B*08:01 is the susceptible haplotype, and C*07:02-B*39:05 and C*07:02-B*39:06 protective haplotypes among Mexican Mestizo SSc patients [21]. 

The association of HLA with diffuse cutaneous systemic sclerosis (dcSSc) and limited cutaneous systemic sclerosis (lcSSc) has been reported from various ethnic groups, and the results were also controversial. A small study from the Netherlands found that HLA *A1, B8* and *DR3* were associated with lcSSc and *DR5(w11)* with dcSSc patients [23]. Another study found that DRB1*11:04, DQA1*05:01 and DQB1*03 were associated with Italian dcSSc and lcSSc patients, whereas DRB1*04:02, DRB1*11:04 and DRB1*15 were associated with Spanish dcSSc patients, and DRB1*08:01, DRB1*11:04, DQA1*04:01, DQB1*04:02 and DQB1*05 with Spanish lcSSc patients [7]. In Asia, DRB1*15:02 and either DRB1*15:02 or DRB1*08:02 were associated with Japanese dcSSc patients [11]. A study from China found that DRB1*11, DRB1*15:02 and DRB1*16 were associated with dcSSc, whereas only DRB1*16 was associated with lcSSc [8]. Among ATA positive patients, DRB1*15:02 and DQB1*06:02 were associated with dcSSc patients, and DRB1*15:02 with Korean lcSSc patients [9]. 

Associations of HLA among dcSSc and lcSSc patients have never been reported in Thailand. Due to the difference in clinical manifestations between dcSSc and lcSSc patients, this study aimed to compare the three-fields level of HLA risk alleles and haplotypes between these two subsets in Thai patients with SSc. The role of the HLA Class I haplotype in the pathogenesis of Thai patients with SSc was also determined. 

## 2. Materials and Methods

In this study, the clinical data and HLA typing results of SSc patients and healthy controls (HCs) were from previously published studies that involved HLA in Thai patients with SSc [12]. In brief, all SSc patients were followed up at the Rheumatology Clinic of Chiang Mai University Hospital, and the diagnosis of SSc met the 1980 American College of Rheumatology classification criteria [24]. Patients overlapping with other connective tissue diseases were excluded. The diagnosis of dcSSc or lcSSc followed the classification of LeRoy et al. [25], and the severity of skin involvement was determined by the modified Rodnan Skin Score [mRSS] [26]. The HCs were unrelated to the patients and had no symptoms or signs suggesting SSc or any other connective tissue diseases. Only one healthy control (HC) was allowed from one family. All of the participants were from northern Thailand, and they provided their written informed consent prior to entering the study. 

HLA alleles were determined for 11 HLA genes (HLA-A, B, C, DRB1, DRB3, DRB4, DRB5, DQA1, DQB1, DPA1 and DPB1) by the Next Generation DNA Sequencing (NGS) method, as described previously [12,27], and genotyped basically to the three-fields level. The Hardy–Weinberg equilibrium (HWE) model was used to determine the distribution of genotype frequency (GF) in the HC population, which was estimated using the AF and number of subjects carrying each allele. 

The HLA haplotypes of the HLA-A, B and C genes and the haplotype frequency (HF) were estimated in SSc patients or HCs by Markov chain–Monte Carlo methods using the PHASE program version 2.1 [28] and at https://stephenslab.uchicago.edu/index.html (assessed on 22 June 2023). The HLA haplotypes and HF among the HLA-DRB1, DQB1 and DPB1 genes, or among alleles from the HLA-A, B, C, DRB1, DQA1, DQB1, DPA1 and DPB1 genes in the overall SSc patient and HC groups, which have been shown in a previous report by the authors [12], are referred to occasionally in the Discussion Section of this study. A family study was not carried out for estimating haplotypes. The linkage disequilibrium (LD) and the relative linkage disequilibrium (RD) were calculated using the HF, which was estimated by the PHASE program and AF of alleles that constituted the haplotype [12]. The significance of LD and RD was not determined. 

The SPSS statistical program version 16.0 (Chicago, IL, USA) was used for statistical analysis. Continuous variables were expressed as the mean ± standard deviation (SD) and the categorical variables were expressed as frequency or percent. Student’s T or Mann–Whitney U tests were used to compare continuous variables. Fisher’s exact test was used for comparing categorical variables including allele frequency (AF). P values were corrected (Pc) by Bonferroni’s method in order to avoid a type I error from multiple comparisons [12,29]. A Pc value of less than 0.05 was considered as a statistically significant difference. The odds ratio (OR) was shown with its 95% confidential interval (95%CI).

## 3. Results

In total, 135 HCs and 92 SSc patients participated in this study. The proportion of female gender was higher in the SSc patients. Of the 92 SSc patients, 55 and 37 were dcSSc and lcSSc patients, respectively. Details of the demographic data among the patients with dcSSc and lcSSc are shown in Table 1. There was no statistically significant difference in age, gender or disease duration. However, the dcSSc patients had a lesser prevalence of hypertension and diabetes mellitus (*p* = 0.022 and *p* = 0.037, respectively). The clinical manifestation between the two groups also was similar, but the dcSSc patients had a significantly higher mRSS score (*p* = 5.727 × 10^−10^), and a higher proportion of those had sclerodactyly (*p* = 2.896 × 10^−6^). In addition, they also had a numerically higher prevalence of ATA (*p* = 0.127) and presence of digital pitting scars (*p* = 0.108), but a lesser prevalence of arthritis (*p* = 0.206). The AF of the HLA genes studied in HCs and dcSSc and lcSSc patients is shown in Table 2**.** The dcSSc patients showed a slight increase in the AF of HLA-A*24:02:01, A*24:07:01, A*74:02:01, B*27:04:01, B*27:06 and C*03:04:01, and also in the AF of B*15:02:01 and C*15:02:01 when compared to the HCs, but without statistical significance. 

Among HLA Class II, the AF of DRB1*15:02:01 (Pc = 0.002) and DRB5*01:02:01 (Pc = 0.044) increased significantly. DRB5*01:08:01N showed an increasing trend, but without statistical significance (Pc = 0.053, OR [95%CI] 2.72 [1.33–5.55]). DRB1*04:05:01 decreased slightly without significance. The AF of DQA1*01:01:01 (Pc = 0.001), DPA1*02:01:01 (Pc = 0.007), DQB1*05:01:24 (Pc = 0.001) and DPB1*13:01:01 (Pc = 1.055 × 10^−4^) increased significantly. DPB1*05:01:01 (Pc = 0.091, OR [95%CI] 0.45 [0.26–0.78]) showed a decreasing trend, but without statistical significance. The AF of DQA1*03:03:01, DPA1*02:02:02 and DQB1*04:01:01 also decreased slightly without significance. 

When comparing lcSSc patients with HCs, the AF of A*24:02:01 (Pc = 0.857, OR [95%CI] 2.44 [1.10–5.44]), A*24:07:01 (Pc = 0.646, OR [95%CI] 4.82 [1.26–18.43]), B*27:04:01 (Pc = 0.377, OR [95%CI] 15.37 [1.69–139.71]) and B*27:06 (Pc = 1.849, OR [95%CI] 5.09 [1.11–23.25]) showed an increasing trend, but not with statistical significance. The AF of C*03:04:01 increased slightly without significance. On the other hand, the AF of A*02:07:01, B*15:02:01 and B*46:01:01 decreased slightly without significance. 

Among HLA Class II, the AF of DRB1*15:02:01 (Pc = 0.271, OR [95%CI] 2.48 [1.31–4.69]), DRB5*01:01:01 (Pc = 0.319, OR [95%CI] 1.93 [1.06–3.50]) and DRB5*01:02:01 (Pc = 0.063, OR [95%CI] 3.60 [1.41–9.22]) showed an increasing trend, but without statistical significance. The AF of DRB1*15:01:01 and DRB5*01:08:01N did not increase significantly. On the other hand, the AF of DRB1*04:05:01 and DRB1*09:01:02 decreased slightly without significance. The AF of DQB1*05:01:24 (Pc = 0.028) and DPB1*13:01:01 (Pc = 6.789 × 10^−4^) increased significantly. DQA1*01:01:01 (Pc = 0.154, OR [95%CI] 2.48 [1.29–4.75]) showed an increasing trend, but without statistical significance. The AF of DPA1*02:01:01 and DPB1*02:02:01 increased slightly without significance. On the other hand, the AF of DPB1*05:01:01 (Pc = 0.002) decreased significantly. The AF of DQA1*03:02:01 and DQB1*03:01 decreased slightly without significance. A rare null allele of HLA-DRB5*01:127N was observed in one lcSSc patient.

The AF values of HLA genes between dcSSc and lcSSc patients were compared and are shown in Table 3. Overall, there was no significant difference in the AF of the HLA Class I and II alleles studied. However, the AF of B*40:06:01 and B*46:01:01 was slightly higher without significance, and the AF of DPB1*02:02:01 slightly lower without significance when comparing dcSSc patients with lcSSc ones. 

The potential HLA Class I and II risk alleles and their associations with clinical features in dcSSc and lcSSc patients are shown in Table 4. Due to the mRSS score and the presence of sclerodactyly being significantly different between the dcSSc and lcSSc patients, only these two clinical parameters were included in the analysis. In using the mRSS score of ≥10, the AF of HLA-A*24:02:01 showed an increasing trend in lcSSc when compared to dcSSc patients (*p* = 0.0254, Pc = not significant (NS), OR [95%CI] 0.17 [0.04–0.72]). The AF of DPA1*02:01:01 also showed an increasing trend in lcSSc with an mRSS score of ≥10 compared to those with a score <10 (*p* = 0.021, Pc = NS, OR [95%CI] 5.61 [1.40–22.56]). The AF of this allele also showed a higher trend in dcSSc than that in lcSSc patients, who had an mRSS score of <10 (*p* = 0.024, Pc = NS, OR [95%CI] 3.57 [1.21–10.57]). The AF of A*24:02:01 in dcSSc patients with sclerodactyly was lower than in those without, and it was also lower than in lcSSc patients with sclerodactyly, but without significance (*p* = 0.061, Pc = NS, OR [95%CI] 0.29 [0.08–1.04] for both conditions). 

The HLA-A, B and C haplotypes with HLA-A*24:02:01, A*24:07:01, A*74:02:01, B*27:04:01, B*27:06 or C*03:04:01, from the top 50 haplotypes in SSc patients and HCs, were listed and are shown in Table 5. Among the top 50 haplotypes in SSc patients, 20 contained at least one of these alleles. Eleven of these alleles were also contained in the top 50 haplotypes in HCs. Among the alleles showing a higher AF in SSc patients, haplotype analysis showed that HLA-A*24:02:01, B*27:06 and C*03:04:01 constituted a haplotype (ABCHP-1), with the fourth most frequent HF in the top 50 haplotypes of HLA-A, B and C (HF = 0.0377). In HCs, the HF of ABCHP-1 that was ≤0.0039 was not included in the top 50 haplotypes. In the top 50 HLA-A, B and C haplotypes, A*02:07:01-C*01:02:01-B*46:01:01 was the most frequent haplotype in SSc patients overall (HF = 0.1002, LD = 0.097, RD = 0.833) and the HCs (HF = 0.1101, LD = 0.105, RD = 0.377), and the HF was almost the same between SSc patients and HCs. The second most frequent haplotype of the top 50 in SSc patients was A*33:03:01-C*03:02:02-B*58:01:01 (HF = 0.0706, LD = 0.070, RD = 0.999). This haplotype was the third most frequent haplotype of the top 50 in the HCs (HF = 0.0592, LD = 0.057, RD = 0.88), with this HF being almost the same as that in the SSc patients. The third most frequent haplotype of the top 50 in the SSc patients was A*11:01:01-C*01:02:01-B*46:01:01 (HF = 0.0378, LD = 0.029, RD = 0.193), which was the sixth most frequent in the HCs (HF = 0.0289, LD = 0.016, RD = 0.096), with the HF being almost the same. The second most frequent haplotype of the top 50 in the HCs was A*11:01:01-C*08:01:01-B*15:02:01 (HF = 0.0608, LD = 0.057, RD = 0.615). This haplotype was the sixth most frequent in the SSc patients (HF = 0.0261, LD = 0.025, RD = 0.587) with a lower HF than that in HCs.

## 4. Discussion

HLA risk alleles among SSc subtypes (dcSSc or lcSSc) have been determined by several authors, but with conflicting results [7,8,9,11,23]. A large study recently used an extensive database from Europe found that *DQA1:05:01* associated with dcSSc, and DQA1*02:01 with lcSSc [6]. A study in the United States involved three ethnic groups (Caucasians, African-Americans and Hispanics) and found that DRB1*11:04, DQB1*03:01, DQB1*26 epitope, DQA1*05:01 and DPB1*13:01 were associated with both dcSSc and lcSSc patients. In addition, DRB1*08 also was associated with dcSSc patients [3]. Another study comprised patients from an Australian cohort and a United States cohort found that DRB1*11:04 was the risk allele for both lcSSc and dcSSc patients, with DPB1*13:01 having the strongest association with dcSSc patients [22]. Moreover, a study from China found that DPB1*03:01, DPB1*13:01 and DPB1*35:01 were associated with both dcSSc and lcSSc patients [30]. 

In this study, the AF of A*24:02:01, A*24:07:01, B*27:04:01 and B*27:06 showed an increasing trend and the AF of C*03:04:01 was slightly higher in lcSSc patients, when compared with the HCs. On the other hand, a slight increase in the AF of these alleles also was seen in the dcSSc patients, but lower than in the lcSSc patients (Table 2). These results suggest that these alleles contribute more to lcSSc than to dcSSc. Unfortunately, a significant increase in B*08:01 or significant decrease in B*44:03, C*07:02 and C*16:01 among the Europeans, Australians and the Mexican Mestizo [6,21,22] was not observed in either dcSSc or lcSSc in the current study. The authors’ previous report found that SSc patients overall tended to have non-significantly higher AF of HLA-A*24:02:01 (Pc = 1.288, OR [95%CI] 2.00 [1.05–3.82]), B*27:04:01 (Pc = 0.974, OR [95%CI] 9.07 [1.08–75.96]) and B*27:06 (Pc = 2.840, OR [95%CI] 4.05 [1.06–15.46]) than the HCs [12]. The AF of A*24:07:01 was slightly higher, but not significantly, in SSc patients overall than in the HCs. 

In terms of haplotype, this study found that A*24:02:01, B*27:06 and C*03:04:01 constituted a common haplotype among Thai patients with SSc. This result was different from those reported from the Mexican Mestizo SSc patients, in that C*07:01-B*08:01 was a susceptible haplotype, and C*07:02-B*39:05 and C*07:02-B*39:06 protective haplotypes [21]. Furthermore, A1, B8 and DR3 reportedly constituted a haplotype that was significantly associated more with lcSSc than dcSSc among the Dutch [23]. These differences might be explained partially by differences in ethnicity. In this study, C*07:01-B*08:01, C*07:02-B*39:05, C*07:02-B*39:06 and A*01-B*08 haplotypes were not observed in the top 50 HLA-A-B-C haplotypes in either the SSc patients overall or HCs. Additionally, no significant difference was observed in the AF of A*01:01:01, B*08:01:01, C*07:01:01 and C*07:02:01 in dcSSc or lcSSc patients in comparison to HCs. B*39:05 and B*39:06 also were not observed in dcSSc and lcSSc patients or HCs. 

The authors’ previous report demonstrated that HLA-DRB1*15:02:01 is linked not only to DRB5*01:02:01, but also to the DRB5*01:08:01N allele [12]. In this study, DRB5*01:02:01 was significantly associated with Thai patients having dcSSc. The trend of DRB5*01:08:01N associating with dcSSc was possibly due to linkage to DRB1*15:02:01, although a family study was not performed (Table 2). In the authors’ previous report, the DRB1*15:02:01, DQB1*05:01:24 and DPB1*13:01:01 alleles were associated with SSc patients (Pc = 7.435 × 10^−4^, 1.433 × 10^−4^ and 9.499 × 10^−7^, respectively). These three kinds of DR, DQ and DP alleles constituted a haplotype (3Hap-1), both in SSc patients overall (HF = 0.1771) and HCs (HF = 0.0763), with the HF being significantly higher in SSc patients overall [12]. The A*24:02:01, B*27:06 and C*03:04:01 alleles, which showed an increasing trend or had a higher frequency in lcSSc in this study, constituted the haplotype ABCHP-1. These three alleles also constituted the eight-locus haplotype (A, B, C, DRB1, DQA1, DQB1, DPA1 and DPB1) with DRB1*15:02:01, DQB1*05:01:24 and DPB1*13:01:01 (8Hap-3). The HF of 8Hap-3 was the third most frequent HF in the top 20 eight-locus haplotypes (HF = 0.0197) in SSc patients, but it was not included in the top 20 eight-locus haplotypes in HCs [12]. 

The above observations suggest that the increasing trend or increase in frequency of the A*24:02:01, B*27:06 or C*03:04:01 alleles observed in lcSSc patients was possibly due to the significant increase in the DRB1*15:02:01, DQB1*05:01:24 or DPB1*13:01:01 alleles. However, the increasing trend in A*24:02:01, B*27:06 and C*03:04:01 was observed mainly in lcSSc patients, despite the small sample size. Although A*24:02:01, B*27:06 and C*03:04:01 did not increase significantly in SSc patients in this study, they possibly played an important role in the pathogenesis of Thai SSc, especially in lcSSc. According to these observations, the possibility of A*24:02:01, B*27:06 and C*03:04:01 contributing genetically to lcSSc, but not dcSSc patients, could not be denied. In order to clarify the contribution of HLA-A, B or C alleles to SSc, further investigation using a large sample size would be necessary. 

On the other hand, these three kinds of DRB1, DQB1 and DPB1 alleles consisted of several eight-locus haplotypes including DQA1*01:01:01 and DPA1*02:01:01 in SSc [12]. In this study, a significant association of both DQA1*01:01:01 and DPA1*02:01:01 with dcSSc was observed (Table 2). The former showed an increasing trend without statistical significance in lcSSc, and the latter showed a non-significant association with lcSSc. Additionally, the increase in DRB1*15:02:01 and DRB5*01:02:01 in lcSSc did not reach statistical significance, although these two alleles increased significantly in dcSSc. The possibility of DQA1*01:01:01, DPA1*02:01:01, DRB1*15:02:01 and DRB5*01:02:01 playing more of a role in the pathogenesis in dcSSc than in lcSSc was suggested. On the other hand, DQB1*05:01:24 and DPB1*13:01:01 were significantly associated with dcSSc and lcSSc, although the significance was higher in dcSSc than in lcSSc. In contrast, the AF of DPB1*05:01:01 was significantly low in lcSSc. DPB1*05:01:01 also decreased in dcSSc patients, but the decrease did not reach statistical significance. Overall, these observations supported a stronger positive contribution of several Class II alleles to dcSSc than to lcSSc [3,6,7,8,9,11,22,30], and they also suggested a stronger protective contribution of DPB1*05:01:01 to lcSSc than to dcSSc. 

In addition, this study suggested that certain HLA alleles might be associated with clinical manifestations among SSc subtypes (Table 4). In those with an mRSS score ≥10, the AF of A*24:02:01 showed a higher trend in lcSSc than in dcSSc. These observations suggested the possibility of A*24:02:01 contributing not only to the pathogenesis of lcSSc (Table 2) but also to a higher mRSS in lcSSc (Table 4). The AF of this allele was lower in dcSSc with sclerodactyly than in dcSSc without, and also lower than in lcSSc with sclerodactyly. These observations suggested the possibility of this allele contributing protectively to sclerodactyly in dcSSc. Similarly, the AF of DPA1*02:01:01 showed a trend to be higher in lcSSc patients with an mRSS score ≥10 than in those with an mRSS score <10. Considering the significant association of this allele with dcSSc, in comparison with the AF of HCs (Table 2), these findings suggested the possibility that DPA1*02:01:01 was not only contributing to the pathogenesis of dcSSc, but also to a higher mRSS in lcSSc (Table 4). Unfortunately, the number of patients in these subgroups was too small to draw conclusions. 

There are some limitations in this study. It was performed from only one study center, with a rather small sample size and no family study. Further studies with a large number of samples are needed to clarify these findings.

Lastly, the non-significant changes in the HWE indicated that the distribution of genotype frequency (GF) in the HCs of this study remained unchanged. In addition, the distribution of AF in most alleles of the HCs in this study and the Thai population from the Allele Frequency Net Database [31] and that from northern Thailand [32] were the same or nearly the same, although there were some alleles that showed a significant difference without correction. Considering the relatively small number of samples, the limited area (near Chiang Mai) where the sampling was carried out in the HCs, the NGS method used for typing and the AFs that were reported in three fields compared to the other two databases which reported in two fields [31,32], the HC data in this study were compatible with the other two databases. 

## 5. Conclusions

This study found that among the HLA Class I molecules, HLA-A*24:02:01, A*27:04:01 B*27:06 and C*03:04:01 showed an increasing trend, but without statistical significance, in Thai lcSSc. A*24:02:01-B*27:06-C*03:04:01 formed a common haplotype in Thai SSc. This haplotype also constituted an eight-locus haplotype of DRB1*15:02:01, DQB1*05:01:24, DPB1*13:01:01, DQA1*01:01:01 and DPA1*02:01:01. In contrast, among HLA Class II, DRB1*15:02:01, DRB5*01:02:01, DQA1*01:01:01, DPA1*02:01:01, DQB1*05:01:24 and DPB1*13:01:01 increased significantly in dcSSc patients, whereas only DQB1*05:01:24 and DPB1*13:01:01 increased in lcSSc patients. DPB1*05:01:01 was protectively associated with lcSSc, but not with dcSSc. These findings suggested that HLA Class I and II might play more roles in the pathogenesis of lcSSc and dcSSc, respectively. Further studies using more samples are needed to confirm and clarify these findings.

## Figures and Tables

**Table 1:** Clinical characteristics of Thai patients studied with SSc and their subtypes.

	Overall SSc (n = 92)	dcSSc (n = 55)	lcSSc (n = 37)	*p* Value (dcSSc vs. lcSSc)
Age, in years (mean ± SD) ^a^	55.32 ± 11.84	53.54 ± 11.95	57.97 ± 11.31	0.078
Female, n (%)	75 (81.52)	44 (80.00)	31 (83.78)	0.647
Disease duration, in years (mean ± SD) ^b^	8.19 ± 6.83	7.85 ± 6.63	8.70 ± 7.19	0.659
Smoking, n (%)	16 (17.39)	10 (18.18)	6 (16.22)	1.000
Alcohol, n (%)	13 (14.13)	7 (12.72)	6 (16.22)	0.762
**Co-morbidities**				
Hypertension, n (%)	16 (17.39)	5 (9.09)	11 (29.73)	0.022
Diabetes mellitus, n (%)	6 (6.52)	1 (1.82)	5 (13.51)	0.037
Dyslipidemia, n (%)	14 (15.22)	8 (14.55)	6 (16.22)	0.827
Chronic kidney disease, n (%)	1 (1.09)	0	1 (2.70)	0.402
Osteoporosis, n (%)	7 (7.61)	2 (3.64)	5 (13.51)	0.113
**Clinical features**				
mRSS score, (mean ± SD) ^b^	12.38 ± 9.82	16.93 ± 9.99	5.62 ± 3.93	5.727 × 10^−10^
Sicca symptoms, n (%)	16 (17.39)	10 (18.18)	6 (16.22)	0.807
Telangiectasis, n (%)	34 (36.96)	20 (36.36)	14 (37.84)	0.886
Raynaud’s phenomenon, n (%)	92 (100.00)	55 (100.00)	37 (100.00)	
Digital pitting scar, n (%)	54 (58.70)	36 (65.45)	18 (48.65)	0.108
Digital gangrene, n (%)	8 (8.70)	4 (7.27)	4 (10.81)	0.555
Sclerodactyly, n (%)	55 (59.78)	44 (80.00)	11 (29.73)	2.896 × 10^−6^
Arthritis, n (%)	28 (30.43)	14 (25.45)	14 (37.84)	0.206
Myositis, n (%)	8 (8.70)	6 (10.91)	2 (5.41)	0.358
Gastrointestinal involvement, n (%)	81 (88.04)	48 (87.27)	33 (89.19)	0.781
Pulmonary fibrosis, n/N (%)	66/87 (75.86)	42/54 (77.78)	24/33 (72.73)	0.593
Pulmonary arterial hypertension, n (%)	22 (23.91)	12 (21.82)	10 (27.03)	0.566
**Serology**				
Anti-nuclear antibody, n (%)	91 (98.91)	55 (97.30)	36 (97.30)	0.402
Anti-topoisomerase-1 antibody, n (%)	63 (68.48)	41 (74.55)	22 (59.46)	0.127
Anti-centromere antibody, n (%)	7 (7.61)	2 (3.64)	5 (15.31)	0.113

a = Student’s T test, b = Mann–Whitney U test. mRSS = modified Rodnan Skin Score, n = number of patients, n/N = number of positive clinical conditions or tests/number of patients tested. Chronic kidney disease ≥ stage III, gastrointestinal involvement including dysphagia, gastro-esophageal reflux disease, diarrhea, constipation, malabsorption and ileus or pseudo-intestinal obstruction.

**Table 2:** Comparison of HLA Class I and II alleles between HCs and dcSSc and lcSSc patients.

Alleles	HCs (n = 135)	dcSSc (n = 55)	Comparison of AF between HC and dcSSc			lcSSc (n = 37)	Comparison of AF between HC and lcSSc		
	AF (2n = 270) #	AF (2n = 110)				AF (2n = 74)			
	%	%	Pc *	OR	95%CI	%	Pc *	OR	95%CI
A*02:07:01	14.81	14.55				8.11			
A*11:01:01	35.56	34.55				28.38			
A*24:02:01	6.67	10.91	NS			14.86	0.857	2.44	1.10–5.44
A*24:07:01	1.48	2.73				6.76	0.646	4.82	1.26–18.43
A*74:02:01	0.37	2.73	1.947	7.54	0.78–73.31	0		ND	
B*13:01:01	8.89	7.27				12.16			
B*15:02:01	9.63	5.45				2.70	2.534	0.26	0.06–1.11
B*27:04:01	0.37	1.82				5.41	0.377	15.37	1.69–139.71
B*27:06	1.11	3.64	4.984	3.36	0.74–15.26	5.41	1.849	5.09	1.11–23.25
B*40:01:02	11.85	10.00				9.46			
B*46:01:01	17.78	20.00				9.46	4.785	0.48	0.21–1.12
C*01:02:01	20.74	21.82				12.16	NS		
C*02:02:02	0	1.82	1.748	ND		0		ND	
C*03:04:01	13.33	14.55				21.62	1.961	1.79	0.93–3.45
C*07:02:01	14.81	14.55				13.51			
C*12:03:01	1.11	3.64	NS			2.70			
C*15:02:01	4.44	0.91	NS			4.05			
DRB1*04:05:01	6.67	1.82	1.994	0.26	0.06–1.14	0	0.492	ND	
DRB1*09:01:02	14.44	12.73				6.76	3.305	0.43	0.16–1.13
DRB1*11:01:01	0.74	0		ND		0		ND	
DRB1*11:06:01	1.48	3.64				0		ND	
DRB1*15:01:01	9.26	8.18				14.86			
DRB1*15:02:01	12.22	30.00	0.002	3.08	1.78–5.32	25.68	0.271	2.48	1.31–4.69
DRB1*16:02:01	11.11	12.73				14.86			
DRB5*01:01:01	17.04	18.18				28.38	0.319	1.93	1.06–3.50
DRB5*01:02:01	3.70	11.82	0.044	3.48	1.48–8.21	12.16	0.063	3.60	1.41–9.22
DRB5*01:03	1.48	1.82				1.35			
DRB5*01:08:01N	6.30	15.45	0.053	2.72	1.33–5.55	10.81	1.445	1.80	0.75–4.36
DRB5*01:127N	0	0		ND		1.35			
DRB5*02:02:01	1.48	0.91				1.35			
DRB5*02:03	3.70	3.64				5.41			
DQA1*01:01:01	11.48	29.09	0.001	3.16	1.81–5.52	24.32	0.154	2.48	1.29–4.75
DQA1*01:05:01	0.00	0.91				2.70	0.870		
DQA1*03:02:01	14.44	12.73				6.76	2.165	0.43	0.16–1.13
DQA1*03:03:01	5.19	0.91	1.466	0.17	0.02–1.29	0	0.885	ND	
DPA1*02:01:01	12.22	26.36	0.007	2.57	1.47–4.50	16.22	2.614	1.39	0.68–7.85
DPA1*02:02:02	55.93	45.45	0.422	0.66	0.42–1.03	55.41			
DQB1*03:01	15.56	10.91				6.76	1.012	0.39	0.15–1.03
DQB1*04:01:01	4.44	0.91	2.156	0.20	0.03–1.54	0		ND	
DQB1*05:01:01	0.37	1.82				2.70			
DQB1*05:01:24	9.63	26.36	0.001	3.36	1.87–6.04	24.32	0.028	3.02	1.55–5.88
DQB1*05:02:01	24.81	27.27				33.78			
DQB1*06:01	7.41	3.64				8.11			
DQB1*06:02:01	2.22	0.91				1.35			
DQB1*06:03:01	0.74	0.91				0		ND	
DPB1*02:02:01	9.63	8.18				17.57	1.344	2.00	0.97–4.12
DPB1*05:01:01	32.96	18.18	0.091	0.45	0.26–0.78	10.81	0.002	0.25	0.11–0.54
DPB1*09:01:01	1.48	1.82				0		ND	
DPB1*13:01:01	15.56	38.18	1.055 × 10^−4^	3.35	2.02–5.56	39.19	6.789 × 10^−4^	3.50	1.98–6.19
DPB1*21:01	4.44	3.64				0	1.615	ND	

# = All alleles in the HCs examined were distributed based on the Hardy–Weinberg equilibrium (HWE). * = Fisher’s P (*p*) values were corrected (Pc) by 26, 45 and 21 for HLA-A, HLA-B and HLA-C, respectively, in the comparison between HCs and dcSSc, and by 26, 45 and 20 for the comparison between HCs and lcSSc. * = Fisher’s P (*p*) values were corrected (Pc) by 27, 5, 3, 6, 19, 6, 18 and 23 for DRB1, DRB3, DRB4, DRB5, DQA1, DPA1, DQB1 and DPB1, respectively, in the comparison between HCs and dcSSc, and by 29, 5, 3, 7, 19, 6, 18 and 21 in the comparison between HCs and lcSSc. Alleles at *p* ≤0.05 or near 0.05 are shown basically. The alleles that were associated with SSc reported previously are also shown. NS = not significant, ND = not done.

**Table 3:** Comparison of HLA Class I and II alleles between dcSSc and lcSSc patients.

Alleles	dcSSc (n = 55)	lcSSc (n = 37)	Comparison of AF between dcSSc and lcSSc		
	AF (2n = 110)	AF (2n = 74)			
	%	%	Pc	OR	95%CI
A*02:07:01	14.55	8.11	5.456	1.93	0.72–5.19
A*11:01:01	34.55	28.38	9.304	1.33	0.70–2.53
A*24:02:01	10.91	14.86	10.943	0.70	0.29–1.69
A*24:07:01	2.73	6.76	5.959	0.39	0.09–1.67
B*13:01:01	7.27	12.16	11.854	0.57	0.21–1.54
B*27:04:01	1.82	5.41	8.640	0.32	0.06–1.82
B*27:06	3.64	5.41	27.920	0.66	0.16–2.73
B*40:01:02	10.00	9.46	39.000	1.06	0.39–2.88
B*40:06:01	4.55	0	3.246	7.77	0.42–142.63
B*46:01:01	20.00	9.46	2.508	2.39	0.97–5.93
C*01:02:01	21.82	12.16	2.587	2.02	0.88–4.63
C*03:04:01	14.55	21.62	5.230	0.62	0.29–1.33
C*07:02:01	14.55	13.51	22.000	1.09	0.47–2.55
C*08:01:01	12.73	5.41	2.876	2.55	0.81–8.09
DRB1*09:01:02	12.73	6.76	4.943	2.01	0.69–5.85
DRB1*11:06:01	3.64	0	3.296	6.30	0.33–118.70
DRB1*15:01:01	8.18	14.86	4.979	0.51	0.20–1.30
DRB1*15:02:01	30.00	25.68	13.574	1.24	0.64–2.41
DRB1*16:02:01	12.73	14.86	14.707	0.84	0.36–1.96
DRB5*01:01:01	18.18	28.38	0.759	0.56	0.28–1.13
DRB5*01:02:01	11.82	12.16	7.000	0.97	0.39–2.40
DRB5*01:08:01N	15.45	10.81	2.741	1.51	0.62–3.70
DQA1*01:01:01	29.09	24.32	7.557	1.28	0.65–2.50
DQA1*01:02:02	15.45	18.92	8.289	0.78	0.36–1.71
DPA1*02:01:01	26.36	16.22	0.739	1.85	0.87–3.92
DPA1*02:02:02	45.45	55.41	1.146	0.67	0.37–1.21
DQB1*05:01:24	26.36	24.32	12.088	1.11	0.57–2.20
DQB1*05:02:01	27.27	33.78	5.767	0.74	0.39–1.39
DQB1*06:01	3.64	8.11	2.859	0.43	0.12–1.57
DPB1*02:02:01	8.18	17.57	1.242	0.42	0.17–1.04
DPB1*05:01:01	18.18	10.81	4.019	1.83	0.76–4.42
DPB1*09:01:01	1.82	0	9.814	3.43	0.16–72.54
DPB1*13:01:01	38.18	39.19	18.210	0.96	0.52–1.76

Fisher’s P (*p*) values were corrected (Pc) by 22, 39, 22, 22, 3, 2, 7, 15, 5, 14 and 19 for HLA-A, HLA-B, HLA-C, DRB1, DRB3, DRB4, DRB5, DQA1, DPA1, DQB1 and DPB1, respectively, in a comparison between dcSSc and lcSSc. Alleles at *p* ≤ 0.05 or near 0.05 are shown basically. Predominant alleles are also shown.

**Table 4:** Comparison of the AF of HLA risk alleles with clinical data in dcSSc and lcSSc patients.

Alleles	mRSS								Sclerodactyly							
	dcSSc (n = 55 Cases)		lcSSc (n = 37 Cases)		Comparison (+ = mRSS ≥10)				dcSSc (n = 55 Cases)		lcSSc (n = 37 Cases)		Comparison (+ = Sclerodactyly Present)			
	AF (2n = 110)		AF (2n = 74)						AF (2n = 110)		AF (2n = 74)					
	≥10	<10	≥10	<10					Positive	Negative	Positive	Negative				
	N of Allele = 78	N of Allele = 32	N of Allele = 12	N of Allele = 62	dc+ vs. dc−	lc+ vs. lc−	dc+ vs. lc+	dc− vs. lc−	N of Allele = 88	N of Allele = 22	N of Allele = 22	N of Allele = 52	dc+ vs. dc−	lc+ vs. lc−	dc+ vs. lc+	dc− vs. lc−
	AF (%)	AF (%)	AF (%)	AF (%)	*p*	*p*	*p*	*p*	AF (%)	AF (%)	AF (%)	AF (%)	*p*	*p*	*p*	*p*
A*24:02:01	7.69	18.75	33.33	11.29	0.104	0.071	0.025 ^a^		7.95	22.73	22.73	11.54	0.061 ^d^	0.285	0.061 ^e^	NS
A*24:07:01	3.85	0	16.67	4.84	0.555	0.183	NS		3.41	0	9.09	5.77	1.000	0.630		
B*27:04:01	1.28	3.13	0	6.45	0.500	1.000			1.14	4.55	0	7.69	0.362	0.311		
B*27:06	2.56	6.25	16.67	3.23	0.578	0.121	0.085		2.27	9.09	4.55	5.77	0.178	1.000		
C*03:04:01	14.10	15.63	33.33	19.35	0.507	0.277	NS		13.64	18.18	27.27	19.23	0.735	0.539	NS	
DRB1*15:02:01	32.05	25.00	41.67	22.58	0.502	0.276			31.82	22.73	18.18	28.85	0.450	0.397	NS	
DRB5*01:02:01	11.54	12.50	16.67	11.29	1.000	0.632			13.64	4.55	13.64	11.54	0.459	1.000		
DQA1*01:01:01	29.49	28.13	33.33	22.58	1.000	0.470		NS	29.55	27.27	22.73	25.00	1.000	1.000		
DQB1*05:01:24	28.21	21.88	33.33	22.58	0.635	0.470			28.41	18.18	22.73	25.00	0.423	1.000		
DPA1*02:01:01	24.36	31.25	41.67	11.29	0.481	0.021 ^b^	NS	0.024 ^c^	23.86	36.36	9.09	19.23	0.281	0.491	NS	NS
DPB1*05:01:01	20.51	12.50	16.67	9.68	0.420	0.608			19.32	13.64	13.64	9.62	0.759	0.688		
DPB1*13:01:01	41.03	31.25	41.67	38.71	0.392	1.000			39.77	31.82	40.91	38.46	0.626	1.000		

Analysis was carried out for Class I alleles showing the trend to be different, and Class II alleles showing a significant difference in comparison between AF in HC and AF in dcSSc and/or lcSSc. Although the AF of C*03:04:01 did not show a different trend, C*03:04:01 was listed because of the linkage with A*24:02:01 and B*27:06. dc = dcSSc, lc = lcSSc, mRSS = modified Rodnan Skin Score. *p* = Fisher’s P value which is shown without correction. Pc, which was corrected by n of comparison (=8), is not shown in the table because all of the Pc values were not significant (NS). a = OR (95%CI): 0.17 (0.04–0.72), b = OR (95%CI): 5.61 (1.40–22.56), c = OR (95%CI): 3.57 (1.21–10.57), d = OR (95%CI): 0.29 (0.08–1.04), e = OR (95%CI): 0.29 (0.08–1.04).

**Table 5:** HLA-A, B and C haplotypes with alleles showing an increasing trend in dcSSc or lcSSc patients.

Code of Haplotype	Haplotypes			HF		LD		RD	
				SSc	HCs	SSc	HCs	SSc	HCs
ABCHP-1	A*24:02:01	C*03:04:01	B*27:06	0.038	≤0.0039	0.037		0.865	
ABCHP-2	A*02:03:01	C*03:04:01	B*13:01:01	0.024	0.018	0.023	0.016	0.249	0.185
ABCHP-3	A*11:01:01	C*03:04:01	B*13:01:01	0.024	0.033	0.018	0.029	0.211	0.339
ABCHP-4	A*11:01:01	C*03:04:01	B*40:01:02	0.018	≤0.0039	0.013		0.137	
ABCHP-5	A*24:02:01	C*03:04:01	B*40:01:02	0.016	≤0.0039	0.014		0.145	
ABCHP-6	A*24:02:01	C*03:04:01	B*13:01:01	0.016	0.007	0.014	0.007	0.154	0.100
ABCHP-7	A*24:02:01	C*14:02:01	B*51:01:01	0.011	≤0.0039	0.011		0.498	
ABCHP-8	A*24:07:01	C*04:01:01	B*35:05:01	0.011	≤0.0039	0.011		0.995	
ABCHP-9	A*11:02:01	C*12:02:02	B*27:04:01	0.010	≤0.0039	0.010		0.306	
ABCHP-10	A*24:07:01	C*04:03:01	B*15:25:01	0.009	≤0.0039	0.009		0.230	
ABCHP-11	A*11:01:01	C*08:01:01	B*27:04:01	0.008	≤0.0039	0.007		0.236	
ABCHP-12	A*24:07:01	C*08:01:01	B*15:02:01	0.008	≤0.0039	0.008		0.174	
ABCHP-13	A*11:01:01	C*12:02:02	B*27:04:01	0.006	≤0.0039	0.005		0.168	
ABCHP-14	A*11:02:01	C*03:04:01	B*13:01:01	0.005	≤0.0039	0.005		0.153	
ABCHP-15	A*11:02:01	C*03:04:01	B*40:01:02	0.005	0.013	0.005	0.012	0.152	0.427
ABCHP-16	A*24:02:01	C*02:02:02	B*27:05:02	0.005	≤0.0039	0.005		0.499	
ABCHP-17	A*24:02:01	C*03:04:01	B*15:01:01	0.005	≤0.0039	0.005		0.489	
ABCHP-18	A*24:02:01	C*14:02:01	B*51:01:02	0.005	≤0.0039	0.005		0.196	
ABCHP-19	A*24:02:40	C*03:04:01	B*13:01:01	0.005	≤0.0039	0.005		0.492	
ABCHP-20	A*74:02:01	C*07:01:01	B*18:01:01	0.005	≤0.0039	0.005		1.000	
ABCHP-21	A*02:07:01	C*03:04:01	B*40:01:02	≤0.0054	0.014		0.012		0.101
ABCHP-22	A*24:02:01	C*01:02:01	B*46:01:01	≤0.0054	0.009		0.007		0.102
ABCHP-23	A*02:03:01	C*03:04:01	B*40:01:02	≤0.0054	0.008		0.006		0.051
ABCHP-24	A*24:07:01	C*01:02:01	B*46:01:01	≤0.0054	0.007		0.007		0.481
ABCHP-25	A*24:02:01	C*03:04:01	B*48:01:01	≤0.0054	0.007		0.007		0.654
ABCHP-26	A*24:02:01	C*07:02:01	B*40:01:02	≤0.0054	0.007		0.006		0.087
ABCHP-27	A*02:01:01	C*03:04:01	B*13:01:01	≤0.0054	0.007		0.006		0.251

Only the top 27 haplotypes are shown in the table. Haplotype frequency was estimated using the PHASE program. Haplotypes with A*24:02:01, A*24:07:01, A*74:02:01, B*27:04:01, B*27:06 or C*03:04:01 were from the top 50 haplotypes of SSc or HCs. Although the AF of C*03:04:01 did not show a different trend, C*03:04:04 was listed because of the linkage to A*24:02:02 and B*27:06. ≤0.0054 and ≤0.0039 means not included in the top 50 SSc and HCs, respectively.

## Data Availability

The datasets used and/or analyzed during the current study are available from the corresponding author on reasonable request due to ethical reason.
